# Supplementary material for: UCSF RMaC: University of California San Francisco 3D Multi-Phase Renal Mass CT Dataset with Tumor Segmentations
Source: medRxiv. 2026 Feb 12:2026.02.11.26346096. Preprint. [Version 1] doi: 10.64898/2026.02.11.26346096 (PMC12919120; doi:10.64898/2026.02.11.26346096)
Supplement: 1 [file NIHPP2026.02.11.26346096V1-supplement-1.pdf]

medRxiv preprint doi: https://doi.org/10.64898/2026.02.11.26346096; this version posted February 12, 2026. The copyright holder for this preprint (which was not certified by peer review) is the author/funder, who has granted medRxiv a license to display the preprint in perpetuity.

It is made available under a CC-BY 4.0 International license .

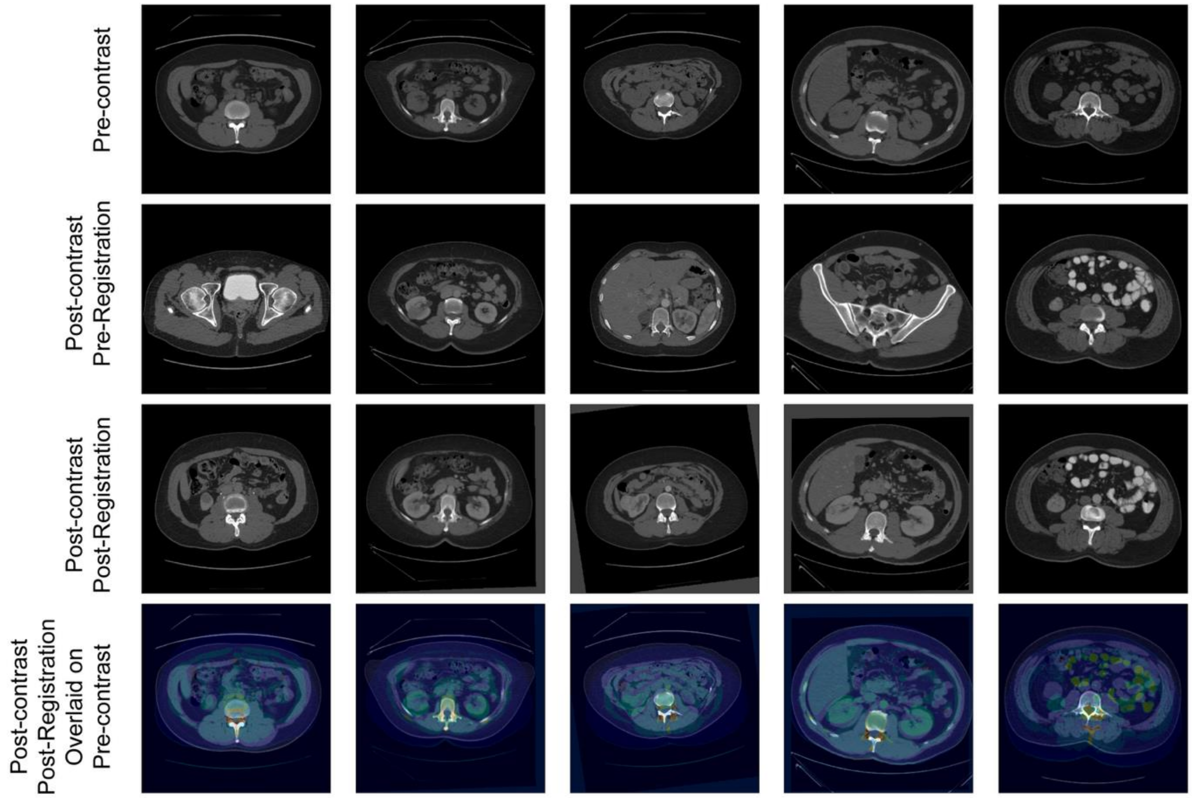

Supplementary Figure: Example of a failed registration. The pre-contrast scan was taken with the patient positioned prone on the CT table and the post-contrast scan here was taken with the patient supine on the table.
